# Evaluation of mitochondrial bioenergetics, dynamics, endoplasmic reticulum-mitochondria crosstalk, and reactive oxygen species in fibroblasts from patients with complex I deficiency

**DOI:** 10.1038/s41598-018-19543-3

**Published:** 2018-01-18

**Authors:** Guilhian Leipnitz, Al-Walid Mohsen, Anuradha Karunanidhi, Bianca Seminotti, Vera Y. Roginskaya, Desiree M. Markantone, Mateus Grings, Stephanie J. Mihalik, Peter Wipf, Bennett Van Houten, Jerry Vockley

**Affiliations:** 1 Division Medical Genetics, Department of Pediatrics, University of Pittsburgh, Pittsburgh, PA 15224 USA; 2 Programa de Pós-Graduação em Ciências Biológicas: Bioquímica, Departamento de Bioquímica, Instituto de Ciências Básicas da Saúde, Universidade Federal do Rio Grande do Sul, Porto Alegre, RS 90035-003 Brazil; 3 Department of Pharmacology and Chemical Biology, University of Pittsburgh, Pittsburgh, PA 15213 USA; 4 Department of Human Genetics, Graduate School of Public Health, University of Pittsburgh, Pittsburgh, PA 15213 USA; 5 Department of Chemistry, University of Pittsburgh, Pittsburgh, PA 15260 USA

## Abstract

Mitochondrial complex I (CI) deficiency is the most frequent cause of oxidative phosphorylation (OXPHOS) disorders in humans. In order to benchmark the effects of CI deficiency on mitochondrial bioenergetics and dynamics, respiratory chain (RC) and endoplasmic reticulum (ER)-mitochondria communication, and superoxide production, fibroblasts from patients with mutations in the *ND6*, *NDUFV1* or *ACAD9* genes were analyzed. Fatty acid metabolism, basal and maximal respiration, mitochondrial membrane potential, and ATP levels were decreased. Changes in proteins involved in mitochondrial dynamics were detected in various combinations in each cell line, while variable changes in RC components were observed. ACAD9 deficient cells exhibited an increase in RC complex subunits and DDIT3, an ER stress marker. The level of proteins involved in ER-mitochondria communication was decreased in ND6 and ACAD9 deficient cells. |ΔΨ| and cell viability were further decreased in all cell lines. These findings suggest that disruption of mitochondrial bioenergetics and dynamics, ER-mitochondria crosstalk, and increased superoxide contribute to the pathophysiology in patients with ACAD9 deficiency. Furthermore, treatment of ACAD9 deficient cells with JP4-039, a novel mitochondria-targeted reactive oxygen species, electron and radical scavenger, decreased superoxide level and increased basal and maximal respiratory rate, identifying a potential therapeutic intervention opportunity in CI deficiency.

## Introduction

Complex I (CI), the largest component of the electron transport chain, is the major entry point of electrons into oxidative phosphorylation (OXPHOS), and catalyzes the transfer of two electrons from NADH^+^ to ubiquinone, thus contributing to the establishment of the proton gradient required for ATP synthesis. It contains 45 subunits forming an “L-shaped” structure with an inner mitochondrial membrane arm and a matrix arm. Fourteen subunits constitute the core of this complex, executing the bioenergetics function. Seven of these core subunits are hydrophobic and encoded by mitochondrial DNA, whereas the other seven are hydrophilic and encoded by the nuclear DNA^1^. ND6, one of the hydrophobic subunits, is localized in the membrane arm of CI and is part of the so called proximal proton pumping module, and NDUFV1 is a hydrophilic polypeptide found in the matrix arm of CI containing the NADH^+^-binding site and the primary electron acceptor FMN^2^.

In addition to the CI components with catalytic and structural functions, CI assembly requires the coordination of several proteins to shuttle nuclear encoded subunits to the mitochondria. Acyl-CoA dehydrogenase 9 (ACAD9), a homodimeric flavoenzyme, is an essential factor for CI assembly. It interacts with other proteins, including NDUFA1, ECSIT, TIMMDC1 and TMEM126B, to form the so-called MCIA complex^3–5^. ACAD9 has also been reported to have long-chain acyl-CoA dehydrogenase activity in tissues where it is highly expressed, such as liver and central nervous system^6,7^.

Isolated deficiency of CI caused by nuclear or mitochondrial DNA mutations is the most commonly identified biochemical defect in childhood-onset mitochondrial OXPHOS disorders, accounting for approximately 40% of all cases^3,8^. CI deficiency is clinically heterogeneous, but the majority of affected individuals develops symptoms during the first year of life and has a rapidly progressive disease course, often resulting in a fatal outcome in childhood. The disease is characterized by hypotonia, seizures, psychomotor retardation, cardiomyopathy, and failure to thrive. Clinical symptoms also include Leigh and Leigh-like syndromes, life threatening infantile lactic acidosis, leukodystrophic encephalopathy, muscle weakness, and developmental delay^9,10^. Milder disease with a predominant myopathy can be seen in adults.

Although the pathophysiology involved in the symptoms observed in patients with mutations in CI subunits has not been fully established, recent studies have revealed an increase of reactive oxygen species (ROS) production, decreased antioxidant defenses^3,11–14^, impairment of the mitochondrial network, and alterations in calcium homeostasis^2,8–10,15–20^. Differential mechanisms of pathophysiology in CI deficiency caused by mutations in nuclear and mitochondrial subunit or assembly factor genes have not been explored.

Alterations in mitochondrial functions have been shown to impair communication of mitochondria with endoplasmic reticulum (ER). These organelles are tightly linked through contact points on their membranes, formed between the voltage-dependent anion channel (VDAC), glucose-regulated protein 75 (Grp75) and inositol 1,4,5-triphosphate receptor (IP3R). This crosstalk has been identified as an important regulator of mitochondrial dynamics, lipid and calcium homeostasis, autophagy and apoptosis^21,22^, and disturbances in it, characterized by either an increase or a reduction of ER-mitochondria interactions, have been reported in several neurodegenerative and metabolic diseases^23–25^.

No effective treatment is currently available to impact the pathophysiology of CI deficiency. Since studies have revealed that oxidative stress is a pathomechanism involved in CI deficiency^11–13^, new therapeutic approaches targeting ROS production have promise. JP4-039 is a synthetic mitochondrial-targeted antioxidant that contains a nitroxide group attached directly to an alkene-peptide isostere. This compound and close analogs have been demonstrated to scavenge ROS and electrons escaping from the respiratory chain (RC), mitigate radiation damage, and prevent lipid peroxidation and apoptosis^26–30^.

This study evaluated the differential effects of mutations in the *NDUFV1*, *ND6* and *ACAD9* genes on various aspects of RC function, endoplasmic reticulum (ER)-mitochondrial communication and ER stress, mitochondrial dynamics, and ROS in fibroblasts of patients with each deficiency. We also investigated the effects of JP4-039, a mitochondrial targeted antioxidant, on ROS generation and mitochondrial respiration in ACAD9 deficient fibroblasts.

## Results

### Protein content of RC components

**Figure 1:**
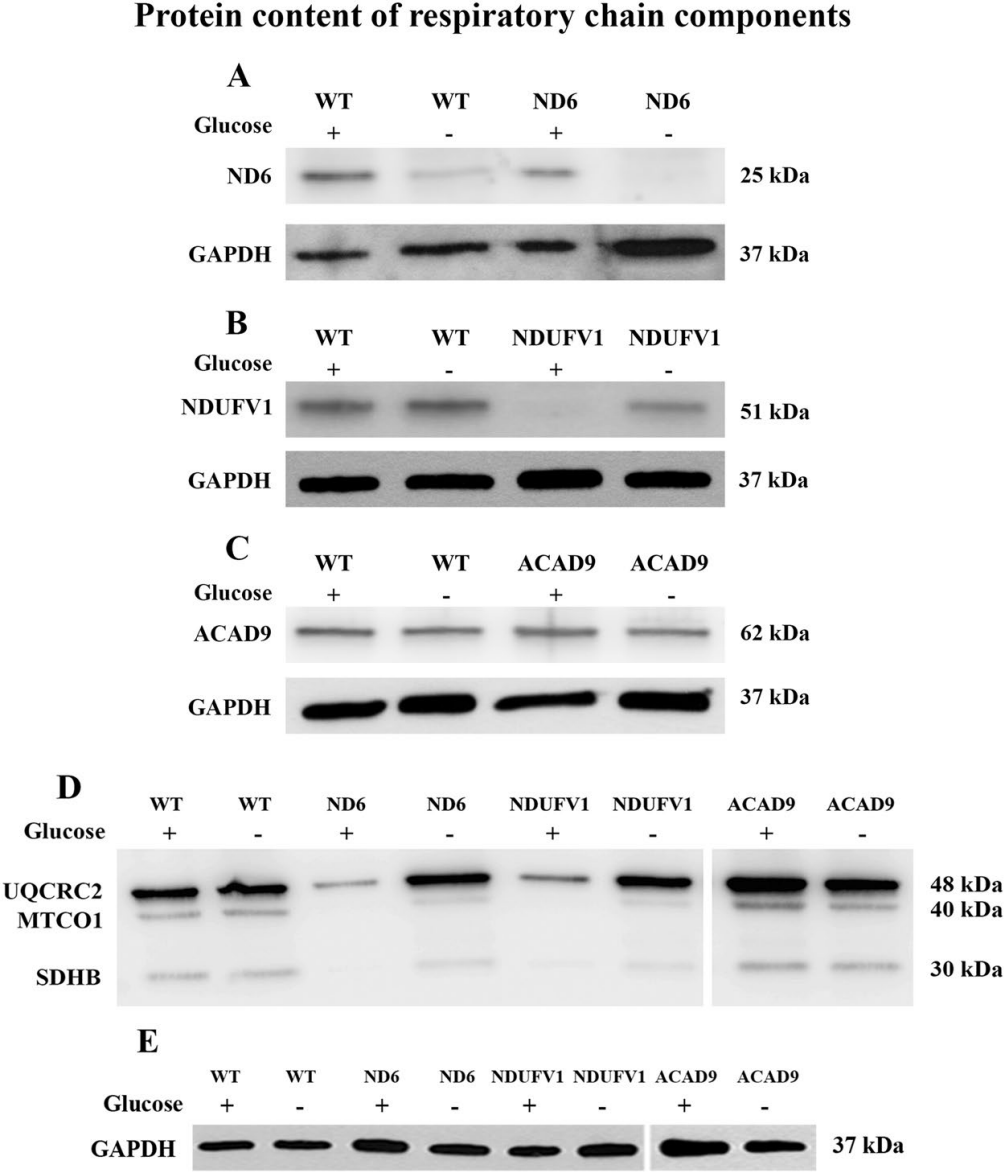
ND6 (complex I subunit) (**A**), NDUFV1 (complex I subunit) (**B**), ACAD9 (complex I assembly factor) (**C**), SDHB (complex II subunit), UQCR2 (complex III subunit) and MTCO1 (complex IV subunit) (**D**) protein content in mitochondria prepared from ND6 deficient, NDUFV1 deficient and ACAD9 deficient fibroblasts cultured in media with or without glucose for 48 h. GAPDH was used as loading control (**A**–**C**, and **E**).

**Figure 2:**
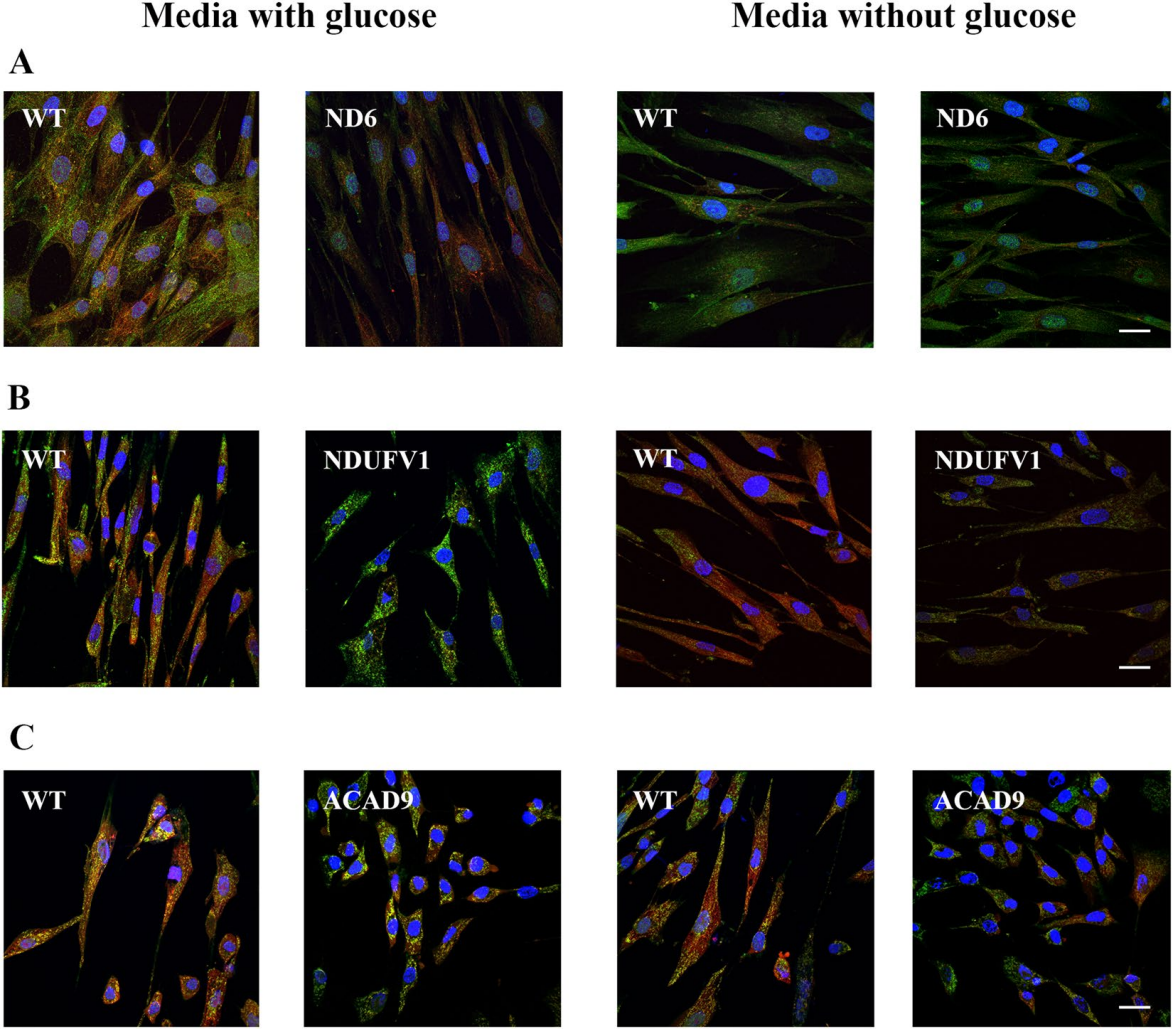
Immunofluorescence analysis of ND6 deficient (**A**), NDUFV1 deficient (**B**) and ACAD9 deficient (**C**) fibroblasts cultured in media with or without glucose for 48 h. The merged image shows colocalization of ND6, NDUFV1 or ACAD9 visualized with green fluorescently tagged antibody and mitochondria visualized with MitoTracker Red as yellow. Nuclei were visualized with DAPI staining.

The quantity of ND6, NDUFV1 or ACAD9 protein in cultured fibroblasts from patients deficient in each gene was evaluated in media with and without glucose after 48 h. Deficiencies of each of these proteins lead to loss of CI activity of the RC. Fibroblasts were grown without glucose to assess the ability of the cells to accommodate the shift of energy source from glycolysis to OXPHOS. ND6 deficient cells had reduced ND6 protein compared to wild type (WT) cells when grown in glucose, and it decreased further when cultured in the absence of glucose (Fig. 1A). NDUFV1 protein also was decreased in patient fibroblasts mutant in this gene grown without glucose and totally absent when glucose was present in the media (Fig. 1B). No difference in ACAD9 protein level was seen in patient fibroblasts mutant in this gene in either condition (Fig. 1C) (Supplementary Table S1). Immunofluorescence studies confirmed the loss of ND6 and NDUFV1 in fibroblasts, and that mutant protein levels were further decreased when glucose was removed from the culture media (Fig. 2).

To determine the effect of the loss of CI on other RC complexes, western blotting of fibroblast extracts with antisera to subunits SDHB (complex II), UQCRC2 (complex III) and MTCO1 (complex IV) was performed. Decreased levels of SDHB and MTCO1 were found in ND6 and NDUFV1 deficient cells as compared to WT cells regardless of the presence of glucose in media. However, UQCRC2 levels were decreased in ND6 and NDUFV1 deficient cells only in the presence of glucose. In ACAD9 deficient cells, SDHB, UQCRC2 and MTCO1 levels were slightly increased under both conditions (Fig. 1D) (Supplementary Table S1).

### Oxygen consumption, mitochondrial membrane potential (ΔΨ), and ATP production

**Figure 3:**
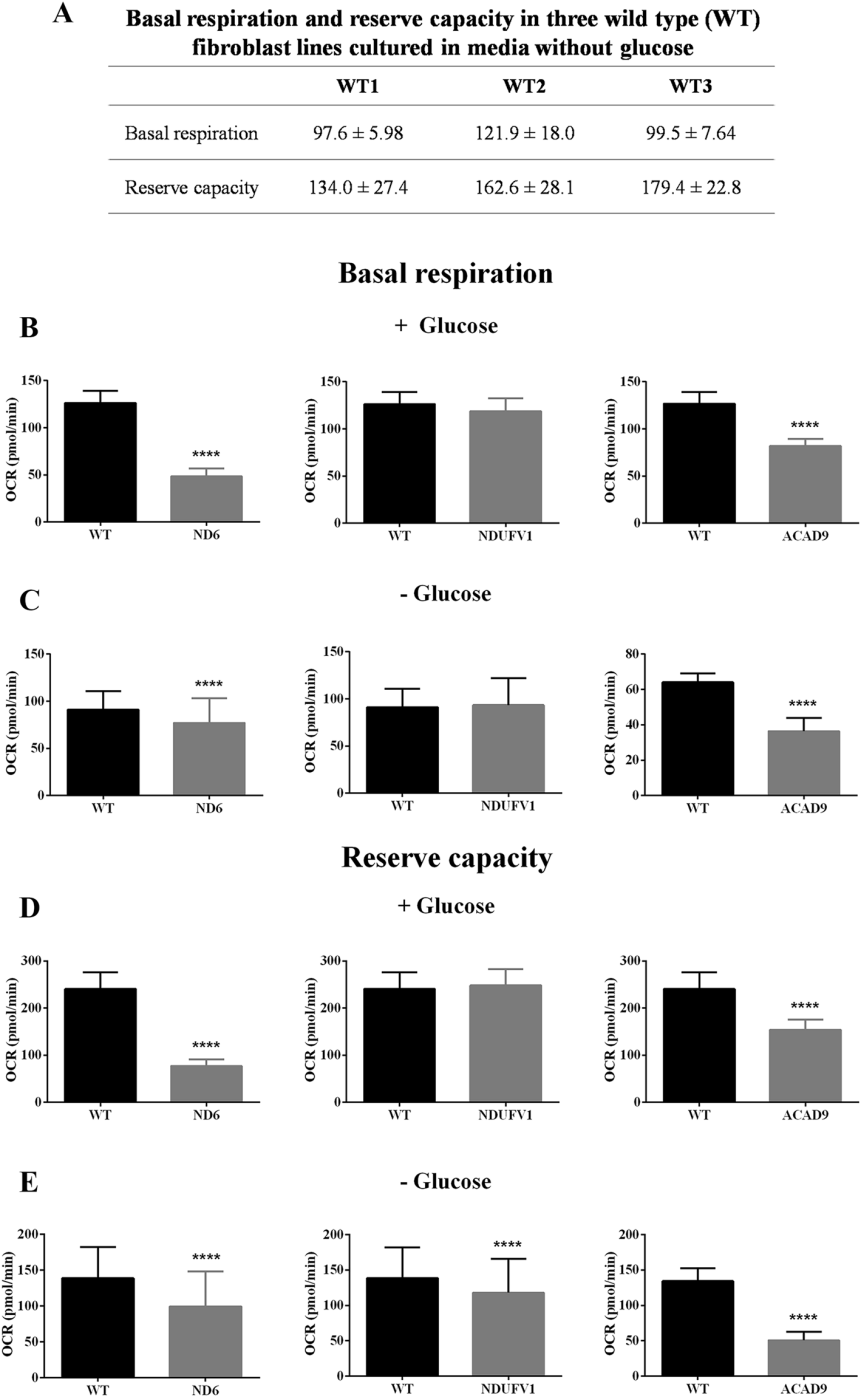
Basal and maximal respiration of three wild type (WT) fibroblast lines (**A**). Basal respiration of ND6 deficient, NDUFV1 deficient and ACAD9 deficient fibroblasts cultured in media with (**B** and **D**) or without glucose for 72 h (**C** and **E**). Data are means ± SD. ****P < 0.0001, compared to wild type (WT) cells (*t* test for unpaired samples) (**B**–**E**).

The bioenergetic status of different WT and patient fibroblasts was assessed by monitoring oxygen consumption in a Seahorse analyzer. Basal and maximal respiration was first analyzed in three different WT fibroblast lines cultured in media without glucose. There was some inherent variation in basal and maximal respiratory rate among control fibroblast lines, so fibroblast WT1 was used for all subsequent experiments (Fig. 3A). In almost all subsequent experiments, the variation between mutant cell lines and WT was greater than among the WT lines. Basal and maximal respiration was decreased in ND6 and ACAD9 deficient cells in media with (Fig. 3B and D) or without (Fig. 3C and E) glucose, whereas NDUFV1 deficient fibroblasts only had a small, but significant, decreased maximal respiration in media devoid of glucose (Fig. 3E).

**Figure 4:**
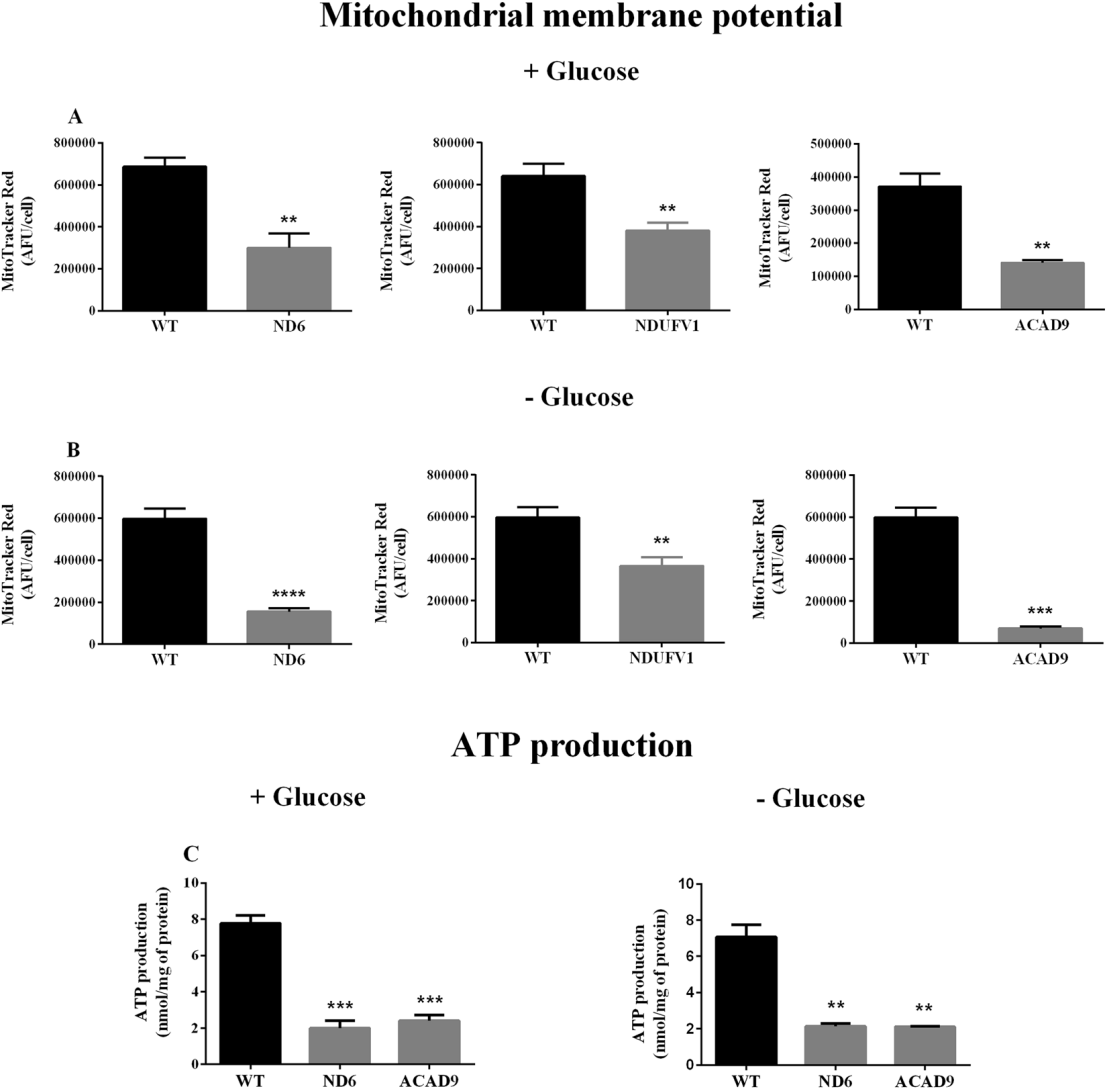
Mitochondrial membrane potential (ΔΨ) (**A** and **B**) and ATP production (**C**) in ND6 deficient, NDUFV1 deficient and ACAD9 deficient fibroblasts cultured in media with or without glucose for 72 h. Data are means ± SD. **P < 0.01, ***P < 0.001, ****P < 0.0001, compared to wild type (WT) cells (*t* test for unpaired samples).

ΔΨ in patient cells was measured using the *in vivo* probe MitoTracker Red to assess the consequences of reduced oxygen consumption. All three cell lines showed a significant reduction of ΔΨ when grown with (Fig. 4A) or without (Fig. 4B) glucose. Consistent with these results, ATP levels in ND6 and ACAD9 deficient cells were markedly reduced (Fig. 4C).

### Fatty acid oxidation (FAO) flux and very long-chain acyl-CoA dehydrogenase (VLCAD) content

**Figure 5:**
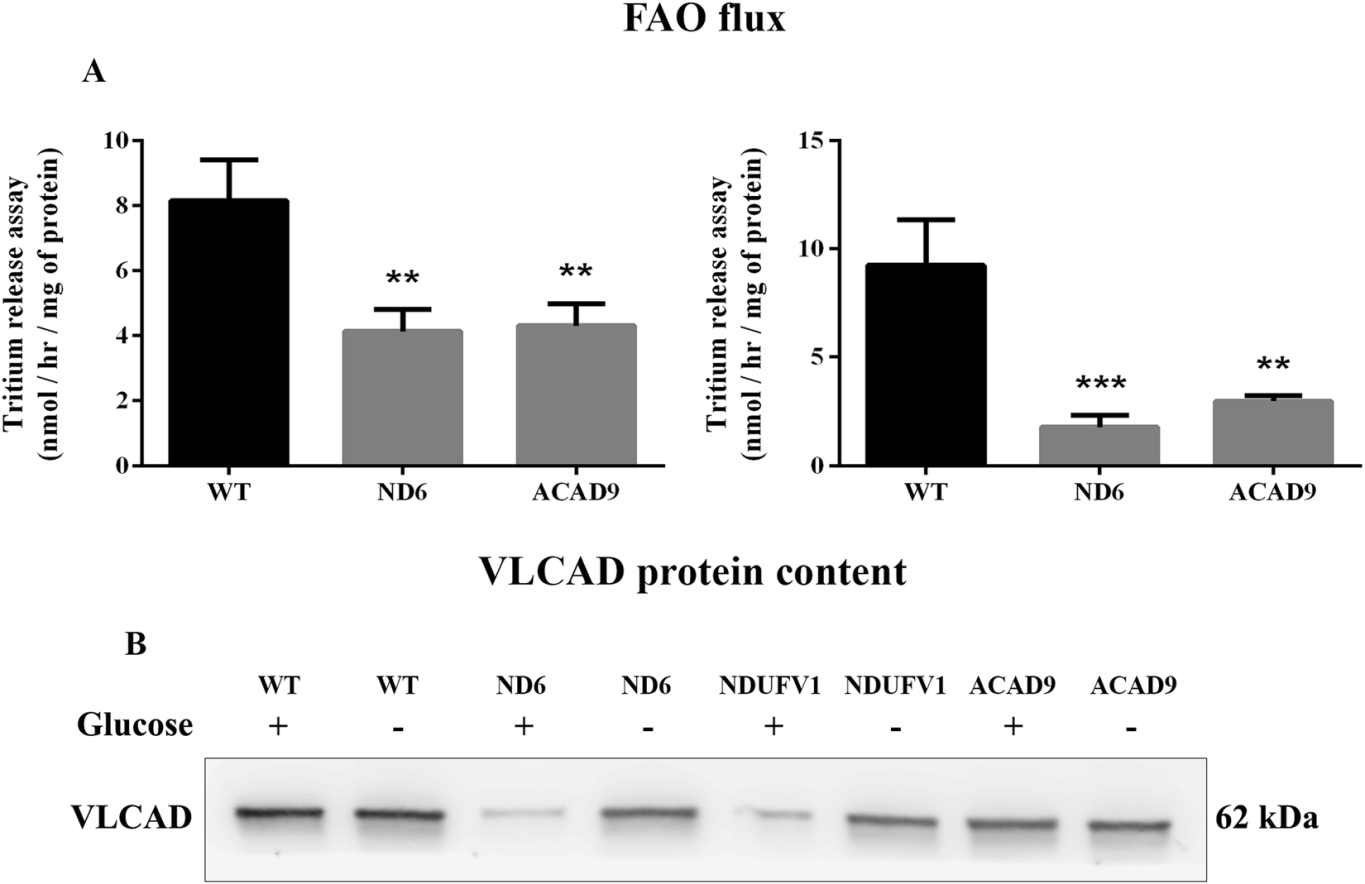
Fatty acid oxidation (FAO) flux (**A**) and very long-chain acyl-CoA dehydrogenase (VLCAD) protein content (**B**) in ND6 deficient, NDUFV1 deficient and ACAD9 deficient fibroblasts cultured in media with or without glucose for 48 h. FAO flux was measured in fibroblasts cultured in a 6-well plate. VLCAD content was measured in mitochondria prepared from fibroblasts. Data are means ± SD. *P < 0.05, compared to wild type (WT) cells (*t* test for unpaired samples).

We have shown that the proteins of FAO and the RC functionally and physically interact in mitochondria and hypothesized that loss of CI might destabilize the complex and affect metabolic flux through FAO in the patient fibroblasts^31^. Decreased oxidation of labeled oleate was indeed seen in ND6 and ACAD9 deficient cells grown with or without glucose (Fig. 5A). VLCAD protein content was decreased in ND6, NDUFV1 and ACAD9 deficient cells, more so in the presence of glucose in the media (Fig. 5B).

### Mitochondrial mass and dynamics

**Figure 6:**
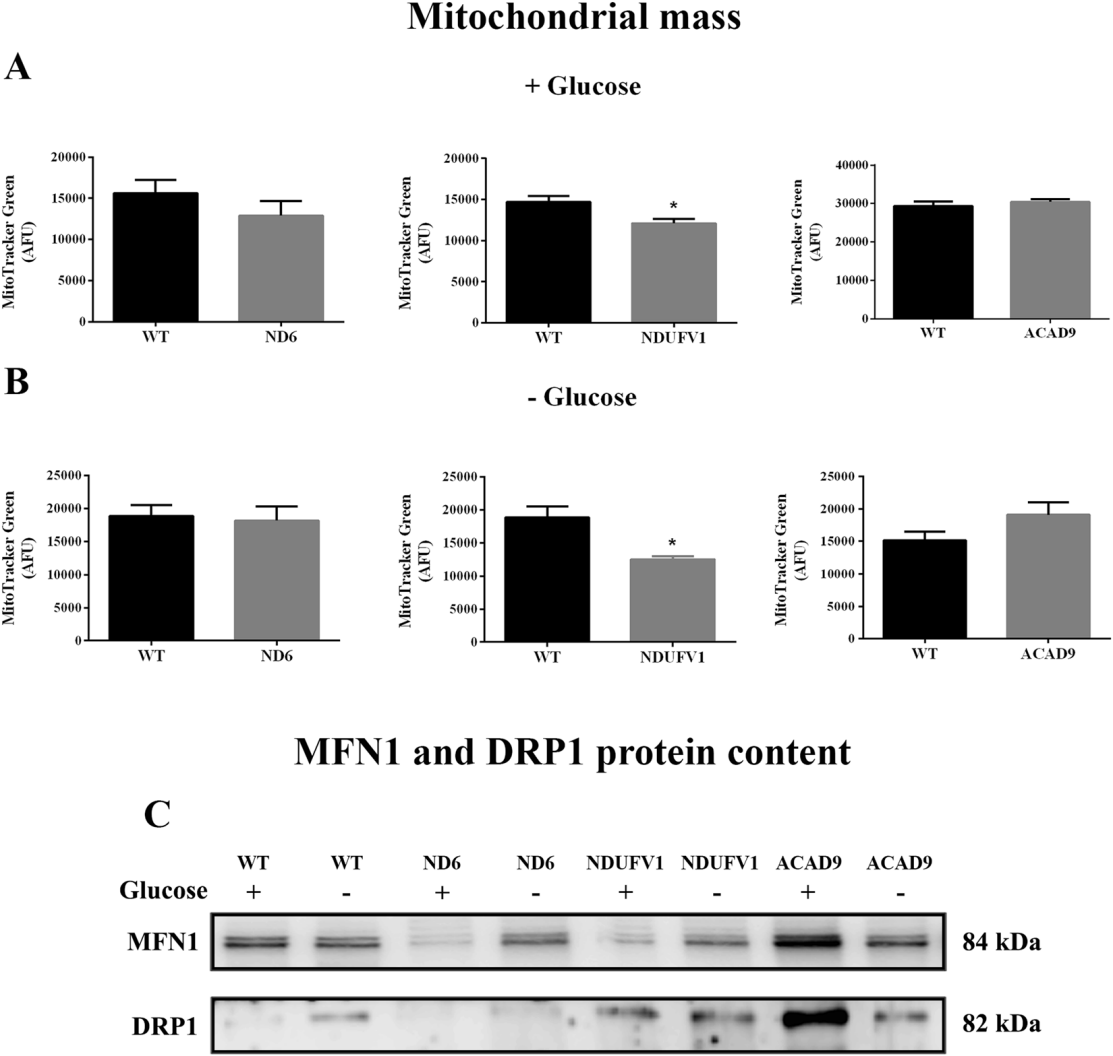
Mitochondrial mass (**A** and **B**) and dynamics (**C**) in ND6 deficient, NDUFV1 deficient and ACAD9 deficient fibroblasts cultured in media with or without glucose for 48 h. For mitochondrial mass, ND6 deficient, NDUFV1 deficient and ACAD9 deficient fibroblasts were incubated with MitoTracker Green. Data are means ± SD. *P < 0.05, compared to wild type (WT) (*t* test for unpaired samples) (**A** and **B**). For mitochondrial dynamics, dynamin-related protein 1 (DRP1) and mitofusin 1 (MFN1) protein content was evaluated in mitochondria prepared from ND6 deficient, NDUFV1 deficient and ACAD9 deficient fibroblasts (**C**).

Mitochondrial mass in patient cells was evaluated using MitoTracker green. Mitochondrial mass was unchanged in ND6 and ACAD9 deficient fibroblasts, but was statistically significantly decreased in NDUFV1 deficient cells in media with or without glucose (Fig. 6A and B). ND6 or ACAD9 deficient fibroblasts were not affected by glucose deprivation (Fig. 6B). Given this result, we measured the levels of MFN1 and DRP1, the main proteins implicated in fusion and fission of mitochondria, respectively (Fig. 6C). DRP1 content was increased in WT cells grown in the absence of glucose compared to growth in glucose without alteration in MFN1 content. DRP1 level was slightly reduced in ND6 deficient fibroblasts grown in media without glucose, while MFN1 was markedly decreased in these cells grown with glucose compared to WT cells. DRP1 was increased in NDUFV1 deficient cells compared to WT in both conditions, while MFN1 was decreased in cells grown in media with glucose. No alterations in MFN1 were observed in ND6 or NDUFV1 deficient cells grown in the absence of glucose. On the other hand, DRP1 and MFN1 were both increased in ACAD9 deficient cells compared to WT in the presence of glucose, but were unaltered in the absence of glucose (Supplementary Table S1).

### Superoxide production

**Figure 7:**
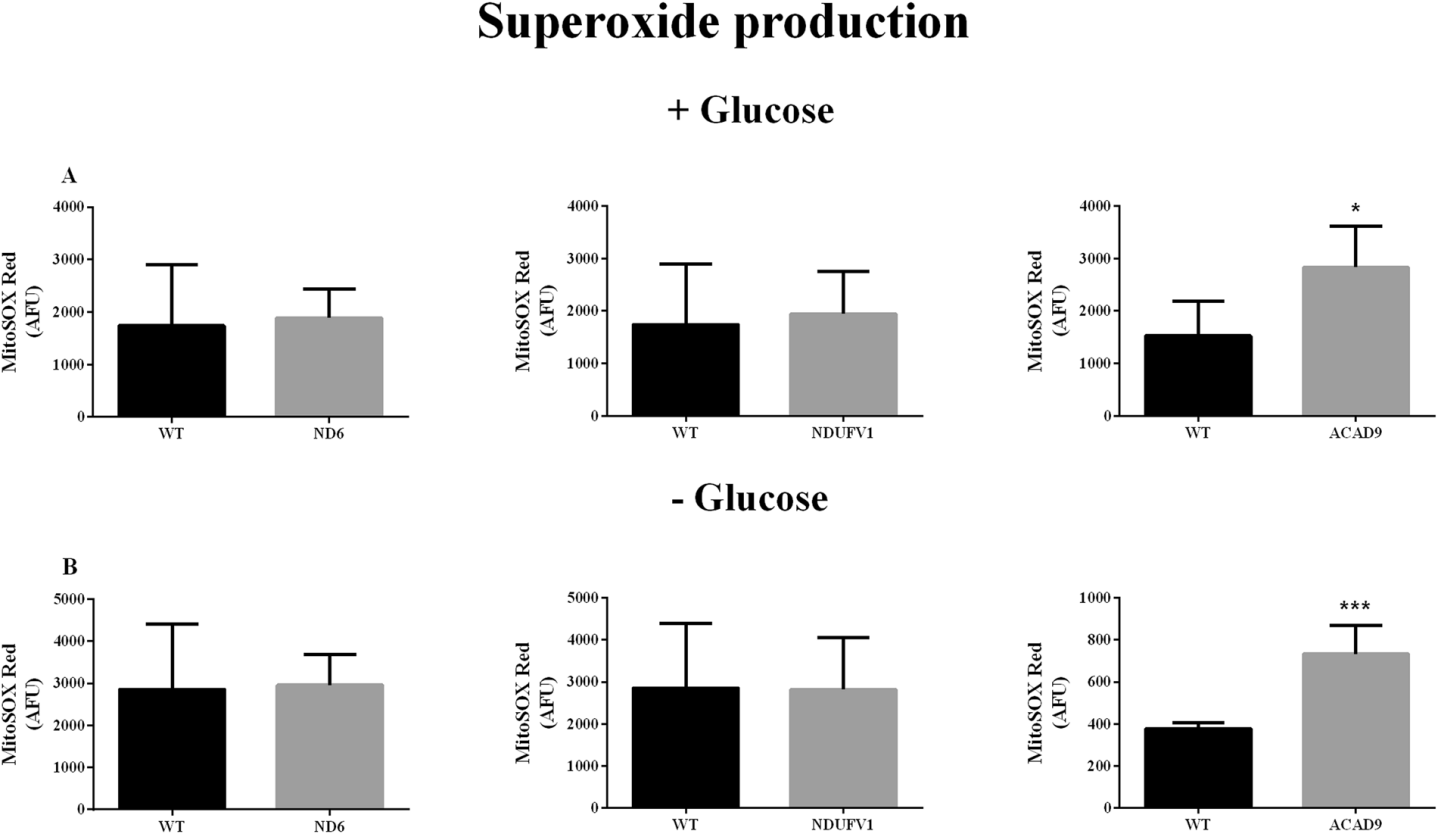
Superoxide production in ND6 deficient, NDUFV1 deficient and ACAD9 deficient fibroblasts cultured in media with (**A**) or without glucose for 48 h (**B**). Data are means ± SD. *P < 0.05, ***P < 0.001, compared to wild type (WT) cells (*t* test for unpaired samples) (**A** and **B**).

Since mitochondrial OXPHOS dysfunction is associated with an increase in ROS, we measured superoxide levels in patient fibroblasts with the molecular probe MitoSOX Red. No significant alterations were observed in ND6 deficient and NDUFV1 deficient cells regardless of the presence of glucose (Fig. 7A and B). In contrast, mitochondrial superoxide production was increased in ACAD9 deficient cells regardless of the presence of glucose (Fig. 7A and B), as normalized to MitoTracker Green (data not shown).

### Protein content of ER-mitochondria crosstalk

**Figure 8:**
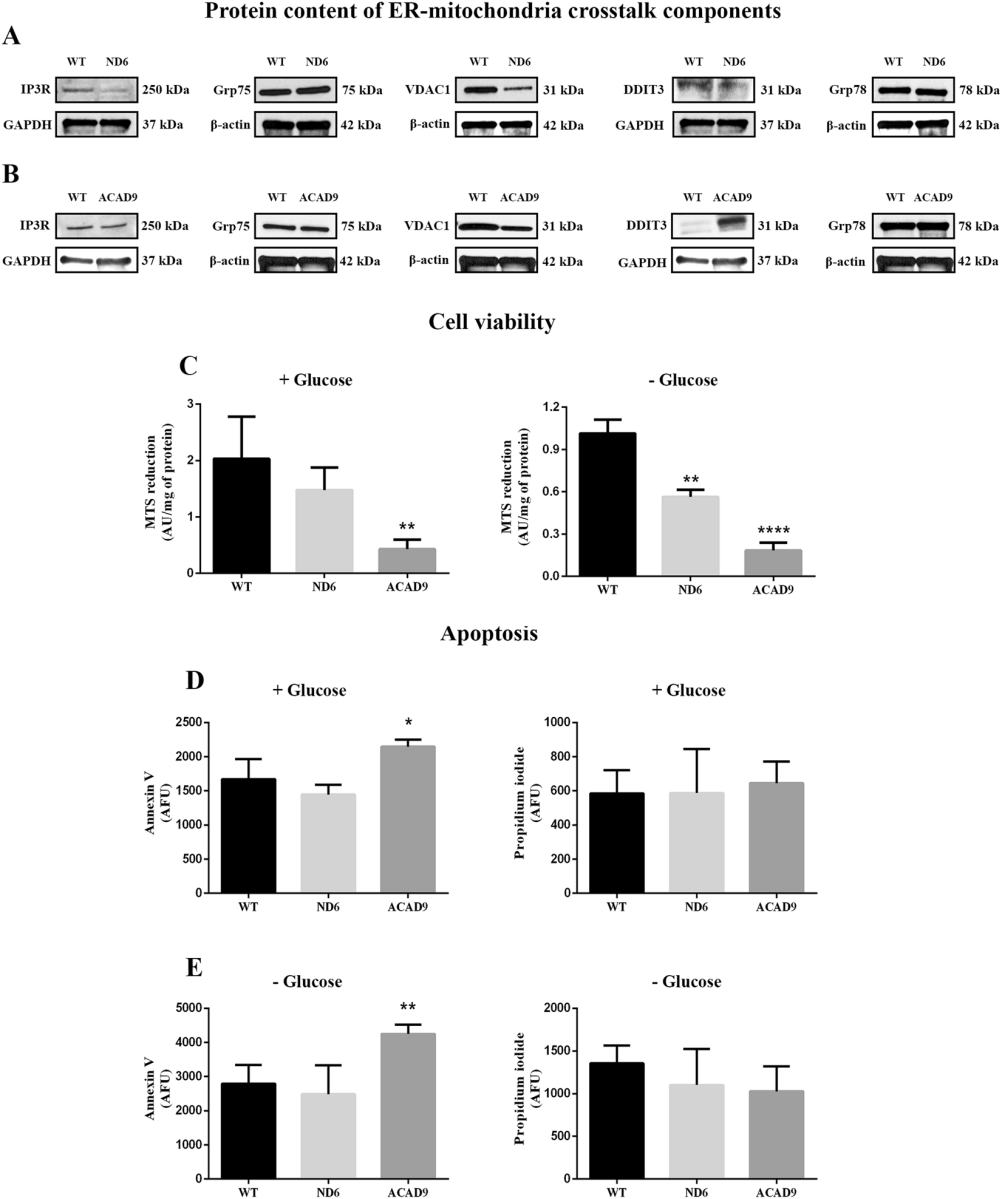
Inositol 1,4,5-trisphosphate receptor 3 (IP3R), glucose-related protein 75 (Grp75), voltage-dependent anion channel 1 (VDAC1), glucose-related protein 78 (Grp78) and DNA damage inducible transcript (DDIT3) protein content in cell lysates prepared from ND6 deficient and ACAD9 deficient fibroblasts cultured in media with glucose Cell viability (**C**) and apoptosis (**D** and **E**) in ND6 deficient and ACAD9 deficient fibroblasts cultured in media with or without glucose for 48 hr. Data are means ± SD. *P < 0.05, **P < 0.01, ****P < 0.0001, compared to wild type (WT) cells (*t* test for unpaired samples).

Alterations in ER-mitochondria crosstalk and ER stress are closely related to mitochondrial dysfunction. Thus, the quantity of proteins involved in this crosstalk (IP3R, VDAC1 and Grp75) and ER stress (DDIT3 and Grp78) were evaluated in ND6 and ACAD9 deficient fibroblasts grown with glucose. VDAC1 was decreased in both cell lines while IP3R was decreased in ND6 deficient cells as compared to WT. Grp75 did not change in either cell line (Fig. 8A and B). Regarding ER stress, DDIT3 was increased in ACAD9 deficient cells, but not in ND6, as compared to WT cells, whereas Grp78 was not altered (Fig. 8B).

### Cell viability and apoptosis

Cell viability of the CI deficient cells was measured with the 3-(4,5-dimethylthiazol-2-yl)-5-(3-carboxymethoxyphenyl)-2-(4-sulfophenyl)-2H-tetrazolium (MTS) reduction assay. Decreased cell viability was seen in ACAD9 deficient fibroblasts grown with or without glucose, whereas a significant reduction of viability was seen in ND6 deficient cells only in media devoid of glucose (Fig. 8C). Since decreased viability may cause cell death, apoptosis was evaluated with the Alexa Fluor® 488 annexin V/Dead Cell Apoptosis kit. Apoptosis was increased in ACAD9 deficient cells in media with or without glucose, but not in ND6, as compared to WT cells (Fig. 8D and E).

### Antioxidant treatment

**Figure 9:**
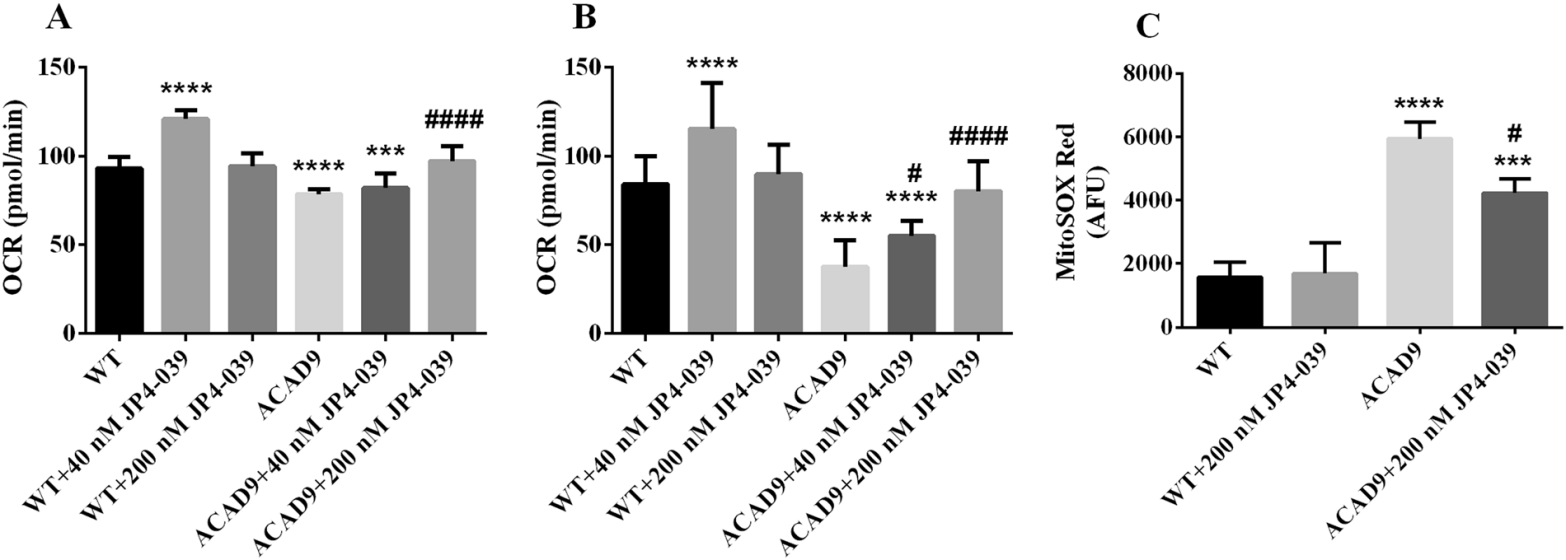
Effect of JP4-039 on basal respiration (**A**), maximal respiration (**B**) and superoxide production (**C**) in ACAD9 deficient fibroblasts cultured in media without glucose for 48–72 h. Cells were exposed to JP4-039 (40 nM or 200 nM) during 24 h. Data are means ± SD. ***P < 0.001, ****P < 0.0001, compared to wilt type (WT) cells; ^#^P < 0.05, ^####^P < 0.0001, compared to ACAD9 deficient cells (Tukey multiple range test).

Increased ROS species has been hypothesized to play a role in inducing damage in cells with impaired RC function. Treatment of ACAD9 deficient cells with the antioxidants N-acetylcysteine, Trolox (a water-soluble vitamin E derivative), mitoQ (ubiquinol), and the pan PPAR promotor transcriptional activator bezafibrate did not reduce, or even increased, superoxide levels (Supplementary Table S2). In contrast, incubation of ACAD9 deficient cells with 40 nM and 200 nM JP4-039, a mitochondrial-targeted antioxidant, significantly increased basal and maximal respiration (Fig. 9A and B) in a concentration dependent fashion, and 200 nM JP4-039 also decreased superoxide level (Fig. 9C) in the absence of glucose.

## Discussion

We have utilized a variety of approaches to evaluate cellular pathophysiology in cultured fibroblasts from healthy individuals and patients affected by CI deficiency. Analysis of mitochondrial respiration with a Seahorse XF analyzer showed some variation in oxygen consumption among WT fibroblast lines. Although we cannot establish the factors responsible for this variation, it may be explained by changes in metabolism, including oxygen consumption, that occur due to aging, as previously reported^32^. Regarding the deficient cell lines, a consistent decrease of basal and maximal respiratory rate in ND6 deficient, NDUFV1 deficient, and ACAD9 deficient cells were observed. The decrease in respiration correlated with ΔΨ dissipation and ATP depletion, with all three parameters indicating that respiratory chain and OXPHOS are markedly impaired in ND6 deficient and ACAD9 deficient cells. Milder alterations were seen in NDUFV1 deficient cells. These data indicate that a Seahorse XF analyzer can reliably demonstrate respiration impairment in a readily available tissue source rather than relying on more difficult to obtain muscle sample, thus allowing analysis of this important class of diseases.

Consistent with functional changes in the RC in patient cells, reduction in RC subunits was found in ND6 and NDUFV1 deficient cells. Although RC subunit protein levels were either unchanged or increased in ACAD9 deficient fibroblasts, suggesting an attempt by cells to compensate for the functional energy deficiency, we and others have previously shown that ACAD9 deficient cells are unable to assemble the CI subunits into active RC supercomplexes^6,14^. Of note, ACAD9 deficient cells exhibited high ROS levels not seen in the other two cell lines suggesting that the cellular derangements in the ACAD9 deficient cells are related at least, in part, to increased ROS levels resulting from failure of CI assembly. In line with this possibility, accumulation of mtDNA deletions in a patient with ACAD9 deficiency has been hypothesized to be related to increased ROS generation^33^.

We have previously reported that the enzymes of FAO exist as part of a multifunctional protein complex within mitochondria that is physically associated with OXPHOS supercomplexes^31^. Moreover, patients with CI deficiency often produce intermediary metabolites suggestive of FAO dysfunction. We therefore tested our CI deficient cell lines for various aspects of FAO function. Whole cell oxidation of radiolabeled oleate, a measure of flux through the entire FAO cycle, was evaluated in ND6 and ACAD9 but not in NDUFV1 deficient cells, while VLCAD content was determined in all cell lines. FAO flux was reduced in ND6 and ACAD9 patient fibroblasts and VLCAD protein was decreased in all cell lines, consistent with impairment of FAO. Thus, these cells demonstrate a compounded bioenergetic impairment. The defect in oleate oxidation was exacerbated by the cellular stress induced by growth in glucose. These findings agree with previous publications showing lipid accumulation in liver and muscle of ACAD9 deficient patients (not a usual finding in most patients with RC deficiencies)^33–35^. Impairment in oleate oxidation in ACAD9 deficient cells has been irregularly reported in the literature, but is consistent when measured under our experimental conditions^6^. Variability in VLCAD content in the various cells implies that multiple mechanisms for dysregulation of the FAO pathway are likely responsible.

Alteration in mitochondrial morphology has been reported in CI deficient cells^18,36,37^, so we evaluated the content of proteins involved in mitochondrial dynamics in patient fibroblasts. A severe dysregulation of fusion and fission is suggested by western blots of MFN1 and DRP1 in ND6 and ACAD9 deficient cells, whereas fission appears to be induced in NDUFV1 deficient cells. Our findings are consistent with previous studies showing that mitochondrial fission occurs when the energy supply is impaired, eliminating damaged mitochondria that produce high levels of ROS^19,38^.

Mitochondrial energy dysfunction has also been shown to disrupt inter-organelle communication between the rough ER and mitochondria and to induce ER stress in cells^25,39,40^. This crosstalk plays a pivotal role in calcium homeostasis, mediating calcium translocation from ER to mitochondria through the interaction of mitochondrial VDAC with IP3R on the ER via the chaperone Grp75^41,42^. In our CI deficient cells, decreased levels of VDAC1 in ND6 and ACAD9 deficient fibroblasts, and of IP3R in ND6 deficient fibroblasts, suggest a disruption of ER-mitochondria crosstalk. A consequent dysregulation of calcium homeostasis could exacerbate bioenergetic failure. Furthermore, ER stress, reflected by increased levels of DDIT3, was seen in ACAD9 deficient cells. Upregulation of DDIT3 corroborates apoptosis increase seen in this cell line since it is a transcription factor that modulates apoptosis through the Bcl-2 family of proteins leading to activation of caspase-3. A large body of evidence has suggested that an excess of reactive oxygen species contributes to damage of the RC in CI deficiency, and that decreasing ROS levels may provide therapeutic value^37,43^. However, use of antioxidants in patients with this disease has been uniformly disappointing, in part, presumably due to poor penetration of the mitochondrial by natural antioxidant supplements. JP4-039 is a mitochondria-targeted ROS and electron scavenger that displays a 20- to 30-fold enrichment in mitochondria over the cytosol in cells^44^. We report here that JP4-039 improved respiration and decreased ROS in ACAD9 deficient fibroblasts, in contrast to traditional antioxidants. Lack of efficacy of a positively charged co-enzyme Q derivative (ubiquinol; mitoQ), which has also been reported to be targeted to mitochondria, may relate to the observation that this compound also binds to non-mitochondrial membranes, and that much of the mitoQ that reaches mitochondria binds to the matrix-facing surface of the inner membrane^45,46^. Thus, only a small fraction remains free in the matrix. Furthermore, charged antioxidants require a functioning mitochondrial transmembrane potential for enrichment.

The most striking difference among the three cell lines was the high levels of ROS identified in ACAD9 deficient fibroblasts. We hypothesize that a defect in assembly of CI induced by ACAD9 deficiency and its broader impact on ETC supercomplexes likely explain this phenomenon^6^. In contrast, isolated CI deficiency represents a less severe defect and therefore leads to less ROS production. This is in line with previous data showing superoxide production is inversely related to CI activity^12^. Indeed, it has been reported that cultured fibroblasts from an NDUFV1 deficient patient can have normal rates of CI activity^47^. It must be acknowledged that abnormalities in fibroblasts are only an approximation of what might be seen in higher energetic tissues. Nonetheless, it is a readily available tissue and, in fact, patient fibroblasts show significant differences from WT, thus providing a tissue that can be readily studied *in vitro*. Moreover, we believe that the variability is less of an issue than the similarities. A larger study will be necessary to make cogent comments about relationship of the various parameters to clinical symptoms.

One aspect of our experimental paradigm is worth further comment. We routinely tested function in cells cultured in media without glucose in order to increase reliance on fatty acids for energy production. This treatment has previously been reported to render cells more sensitive to mitochondrial toxicants^48^. Therefore, we anticipated, and indeed saw, that culture in glucose free media would accentuate mitochondrial derangements caused by CI deficiency. The |ΔΨ| was uniformly further decreased in all cell lines cultured in the absence of glucose, as was cell viability. Interestingly, each of the cell lines exhibited some differential effects, such as changes in the content of RC components and DRP1. The reason for these differences remains to be elucidated and may ultimately be useful in identifying disease specific therapies. It is difficult to project the pathophysiological significance of our fibroblast findings onto other tissues affected in CI deficiency such as central nervous system and heart. However, it is tempting to speculate that the cellular derangements identified in the present study will be magnified in these tissues due to their greater number of mitochondria and reliance on OXPHOS for energy.

In summary, we have used a wide variety of techniques to demonstrate the effects of mutations affecting CI of the RC at the cellular level in a tissue that is readily accessible from patients. We show that the bioenergetic failure in this disorder extends to other energy deriving pathways, and predict that changes in mitochondrial dynamics, ER-mitochondria communication, and accumulation of reactive species are all likely to play a role in CI deficiency. Finally, we report that treatment of fibroblasts with impairment in CI due to ACAD9 deficiency with the mitochondrial-targeted ROS scavenger JP4-039 corrected their bioenergetic defect and decreased ROS levels, identifying this molecule as a promising candidate drug for treatment of this disorder.

## Methods

Methods were carried out in accordance with the approved guidelines and regulations. Experimental protocols were approved by the Institutional Review Board at the University of Pittsburgh, protocol #404017.

### Subjects

Patient skin biopsies for fibroblast culture were performed on a clinical basis with written informed consent from patients and/or legal guardians. Fibroblast cells with mutations in the *ND6*, *NDUFV1*, or *ACAD9* gene were obtained from patients’ skin biopsies, while wild type (WT) fibroblast cells were obtained from healthy individuals.

Biopsies from patients were performed on a clinical basis with written informed consent from patients and/or parents, or from anonymous donors. The NDUFV1 and ACAD9 deficient patients were previously reported^6,47,49^. The ND6 deficient patient had a heteroplasmic mutation (65% mutant) on mtDNA at position 14459. The NDUFV1 deficient patient was compound heterozygous for two exon 5 mutations: c.611A>G (p.Y204C), and c.616T>G (p.C206G). The ACAD9 patient was homozygous for a c.1553G>A (p.R518H) mutation. Clinical signs and mutations are described in Supplementary Table S3.

### Cell culture and treatments

Cells were grown in Dulbecco’s Modified Eagle Medium (DMEM), Corning Life Sciences, Manassas, VA, containing high glucose levels (4.5 g/L) or in DMEM devoid of glucose for 48–72 hr. Both media were supplemented with 10% fetal bovine serum, 4 mM glutamine, 100 IU penicillin and 100 μg/mL streptomycin, Corning Life Sciences, Manassas, VA. In some experiments, ACAD9 deficient fibroblasts were incubated for 24 hr with JP4-039 (40 or 200 nM), obtained from Dr. Peter Wipf, Department of Chemistry, University of Pittsburgh^50^, N-acetylcysteine (1 mM), Sigma-Aldrich Co., St. Louis, MO, resveratrol (5 µM), Sigma-Aldrich Co., St. Louis, MO, mitoQ (200 nM), MitoQ Limited, Auckland, New Zealand, Trolox (a hydrosoluble analogue of vitamin E; 1 mM), Sigma-Aldrich Co., St. Louis, MO, or bezafibrate (600 µM), Sigma-Aldrich Co., St. Louis, MO, prior to the analysis of parameters.

### Western blotting

Cells were grown in T175 flasks to 90–95% confluence, then harvested by trypsinization, pelleted, and stored at −80 °C for western blot. Cell pellets were used to prepare cell lysates or mitochondria. For cell lysates, pellets were re-suspended in 150–250 μL of RIPA buffer with protease inhibitor cocktail, Roche Diagnostics, Mannheim, Germany. Homogenates were kept on ice for 30 min, shaken every 10 min, and centrifuged at 14,000 × *g* for 15 min at 4 °C. Supernatants were used for western blotting. For mitochondria, pellets were re-suspended in 150–250 μL of 5 mM Tris buffer, pH 7.4, containing 250 mM sucrose, 2 mM EDTA, protease inhibitor cocktail, Roche Diagnostics, Mannheim, Germany, and 0.5 μM trichostatin A, Sigma-Aldrich Co., St. Louis, MO, homogenized and centrifuged at 1,000 × *g* for 5 min at 4 °C. The pellet was discarded and the supernatant centrifuged at 12,000 × *g* for 15 min at 4 °C. The resulting pellet containing mitochondria was re-suspended in 50 mM Tris buffer, pH 7.4, sonicated and centrifuged again at 14,000 × *g* for 15 min at 4 °C.

Cell lysates or mitochondria were used for western blotting as previously described^16^. Briefly, 10 or 20 μg of protein were loaded onto the gel. Following electrophoresis, the gel was blotted onto a nitrocellulose membrane, which was incubated with rabbit anti-ND6 polyclonal antibody (1:100), Santa Cruz Biotechnology, Dallas, TX, rabbit anti-NDUFV1 polyclonal antibody (1:100), Santa Cruz Biotechnology, Dallas, TX, rabbit anti-ACAD9 antiserum (1:500), Cocalico Biologicals Inc., PA, rodent anti-total OXPHOS cocktail antibody (1:250), Abcam, Cambridge, MA, mouse anti-mitofusin 1 (MFN1) monoclonal antibody (1:100), Abcam, Cambridge, MA, mouse anti-dynamin-related protein 1 (DRP1) monoclonal antibody (1:100), Abcam, Cambridge, MA, rabbit anti-very long-chain acyl-CoA dehydrogenase (VLCAD) antiserum (1:1,000), Cocalico Biologicals Inc., PA, rabbit anti-voltage-dependent anion channel 1 (VDAC1) monoclonal antibody (1:1,000), Abcam, Cambridge, MA, mouse anti-glucose-related protein 75 (Grp75) monoclonal antibody (1:250), Abcam, Cambridge, MA, rabbit anti-glucose-related protein 78 (Grp78) polyclonal antibody (1:250), Abcam, Cambridge, MA, mouse anti-DNA damage inducible transcript 3 (DDIT3) monoclonal antibody (1:250), Abcam, Cambridge, MA, goat anti-inositol 1,4,5-trisphosphate receptor 3 (IP3R) polyclonal antibody (1:50), Santa Cruz Biotechnology, Dallas, TX, or IgG-HRP conjugated antibody, Bio-Rad, Hercules, CA. Staining of the membranes with Ponceau S, Sigma-Aldrich Co., St. Louis, MO, or mouse anti-β-actin monoclonal antibody (1:10,000), Sigma-Aldrich Co., St. Louis, MO, or mouse anti-glyceraldehyde 3-phosphate dehydrogenase **(**GAPDH) monoclonal antibody (1:15,000), Abcam, Cambridge, MA, was used to verify equal loading. Gel images from all figures conform to the journal policy as noted at www.nature.com/srep/policies/index.html#digitalimage, http://www.nature.com/srep/policies/index.html#digital-image. Images were electronically adjusted to optimize comparisons within a single gel but not for comparisons across different gels. High contrast and overexposed images were not utilized.

### Measurement of mitochondrial respiration

Oxygen consumption rate (OCR) was measured with a Seahorse XF^e^96 Extracellular Flux Analyzer, Seahorse Bioscience, Billerica, MA. The apparatus contains a fluorophore that is sensitive to changes in oxygen concentration, which enables it to accurately measure the rate at which cytochrome *c* oxidase (complex IV) reduces one O_2_ molecule to two H_2_O molecules during OXPHOS. Cells were seeded in 96-well Seahorse tissue culture microplates in growth media at a density of 80,000 cells per well. To ensure equal cell numbers, cells were seeded in cell culture plates pre-coated with Cell-Tak, BD Biosciences, San Jose, CA. All cell lines were measured with four to eight wells per cell line. Then, the entire set of experiments was repeated. Before running the Seahorse assay, cells were incubated for 1 h without CO_2_ in unbuffered DMEM. Initial OCR was measured to establish a baseline (basal respiration). Maximal respiration was also determined after the injection of 300 nM carbonyl cyanide 4-(trifluoromethoxy)phenylhydrazone (FCCP), Seahorse XF Cell Mito Stress Test Kit, Santa Clara, CA. Data were reported in pmol/min for OCR.

### Mitochondrial membrane mass and superoxide production

Cell suspensions containing 1 × 10^5^ cells/mL were incubated for 25 min at 37 °C with 150 nM MitoTracker Green, Invitrogen, Grand Island, NY, for mitochondrial mass evaluation, or for 15 min at 37 °C with 5 µM MitoSOX Red, Invitrogen, Grand Island, NY, for superoxide production measurement. After incubation, 10,000 cells were analyzed in a Becton Dickinson FACSAria II flow cytometer, BD Biosciences, San Jose, CA.

### Immunofluorescence microscopy and mitochondrial membrane potential (ΔΨ)

Fibroblasts were seeded at a concentration of 1 × 10^5^ cells/mL on glass cover slips pre-treated with poly-D-lysine in a 12-well plate and allowed to grow overnight at 37 °C in a 5% CO_2_/95% humidity incubator. At 80–90% confluence, cells were incubated with 350 nM MitoTracker Red, from Invitrogen, for 30 min at room temperature, washed 3 times with TBST buffer and fixed with 4% paraformaldehyde for 30 min. This was followed by incubation with the antibodies anti-NDUFV1 (1:50), anti-ND6 (1:100) or anti-ACAD9 (1:1,000) at 4 °C overnight. After brief washing with TBST, cells were incubated with donkey anti-rabbit secondary antibody Alexa Fluor 488, from Invitrogen. Nuclei were immunostained with DAPI. The coverslips were then mounted using mounting media before taking images with an Olympus Confocal FluoroView1000 microscope at a magnification of 60×. Mitochondrial membrane potential was determined by quantification of MitoTracker Red fluorescence, using the software ImageJ, Bethesda, MD, and the data were normalized by number of cells.

### ATP production assay

ATP production was determined by a bioluminescence assay using an ATP determination kit (ATPlite kit) from PerkinElmer Inc, Waltham, MA, according to the manufacturer’s instructions. The luminescence was measured in a FLUOstar Omega plate reader, BMG Labtech, Ortenberg, Germany.

### Fatty acid oxidation (FAO) flux analysis

Tritium-release assay was performed according to the method of Bennett^51^. Specifically, 300,000 fibroblasts were plated per well in 6-well plates and grown for 24 hr in DMEM with 10% fetal bovine serum. The growth media was then changed to either the same media or devoid of glucose and fibroblasts were grown as described for 48 hr. Subsequently, cells were washed once with PBS and then incubated with 0.34 µCi [9,10-³H]oleate (45. 5 Ci/mmol; Perkin Elmer, Waltham, MA) in 50 nmol of oleate prepared in 0.5 mL glucose-free DMEM with 1 µg/ml carnitine and 2 mg/ml α-cyclodextrin for 2 hr at 37 °C. Fatty acids were solubilized with α-cyclodextrin as described^52^. After incubation, ^3^H_2_O released was separated from the oleate on a column containing 750 µL of anion exchange resin (AG 1 × 8, acetate, 100–200 Mesh, BioRad, Richmond, CA) prepared in water. After the incubation medium passed through the column, the plate was washed with 750 μL of water which was also transferred to the column. The resin was then washed twice with 750 μL of water. All eluates were collected in a scintillation vial and mixed with 5 mL of scintillation fluid (Eco-lite, MP), followed by counting in a Beckman scintillation counter in the tritium window. Assays were performed in quadruplicate with triplicate blanks (cell free wells). Standards contained a 50 µL aliquot of the incubation mix with 2.75 mL of deionized water and 5 mL of scintillation fluid.

### Cell viability assay

Cell viability was evaluated with a 3-(4,5-dimethylthiazol-2-yl)-5-(3-carboxymethoxyphenyl)-2-(4-sulfophenyl)-2H-tetrazolium (MTS) assay kit according to the manufacturer’s instructions, Abcam, Cambridge, MA. The absorbance was read in the FLUOstar Omega plate reader at 490 nm.

### Apoptosis assay

Apoptosis was evaluated with an Alexa Fluor® 488 annexin V/Dead Cell Apoptosis kit according to manufacturer’s instructions, Invitrogen, Grand Island, NY. The kit contains annexin V labeled with a fluorophore and propidium iodide (PI). Annexin V can identify apoptotic cells by binding to phosphatidylserine exposed on the outer leaflet of cell plasma membrane while PI stains dead cells by binding to nucleic acids. Fluorescence was determined in a Becton Dickinson FACSAria II flow cytometer, BD Biosciences, San Jose, CA.

## Electronic supplementary material

Supplementary Tables
