# Supplementary material for: Evaluation of mitochondrial bioenergetics, dynamics, endoplasmic reticulum-mitochondria crosstalk, and reactive oxygen species in fibroblasts from patients with complex I deficiency
Source: Sci Rep. 2018 Jan 18;8:1165. doi: 10.1038/s41598-018-19543-3 (PMC5773529; doi:10.1038/s41598-018-19543-3)
Supplement: Supplementary file 1 — Supplementary Tables [file 41598_2018_19543_MOESM1_ESM.pdf]

SUPPLEMENTARY INFORMATION

Title of the manuscript: Evaluation of mitochondrial bioenergetics, dynamics, endoplasmic reticulum-mitochondria crosstalk, and reactive oxygen species in fibroblasts from patients with complex I deficiency

Authors: Guilhian Leipnitz, Al-Walid Mohsen, Anuradha Karunanidhi, Bianca Seminotti, Vera Y. Roginskaya, Desiree M. Markantone, Mateus Grings, Stephanie J. Mihalik, Peter Wipf, Bennett Van Houten, Jerry Vockley

Supplementary Table S1. Protein content of respiratory chain components, mitofusin1 (MFN1) and dynamin-related protein 1 (DRP1) in mitochondria prepared from ND6 deficient (ND6d), NDUFV1 deficient (NDUFV1d) and ACAD9 deficient (ACAD9d) fibroblasts. Fibroblasts were cultured in media with or without glucose for 48 h. (+) means increased content, (–) means decreased content, (=) means similar content, compared to wild type cells; ND means not done.

Respiratory chain component	ND6d Media with glucose	ND6d Media without glucose	NDUFV1d Media with glucose	NDUFV1d Media without glucose	ACAD9d Media with glucose	ACAD9d Media without glucose
ND6	–	–	**ND**	**ND**	**ND**	**ND**
NDUFV1	**ND**	**ND**	–	–	**ND**	**ND**
ACAD9	**ND**	**ND**	**ND**	**ND**	=	=
UQCRC2	–	=	–	=	+	+
MTCO1	–	–	–	–	+	=
SDHB	–	–	–	–	+	+
MFN1	–	=	–	=	+	=
DRP1	=	–	+	+	+	=

Supplementary Table S2. Effect of protective compounds on superoxide production in ACAD9 deficient (ACAD9d) fibroblasts cultured in media without glucose for 48 hr.

	ACAD9d	ACAD9d + protective compound
N-acetylcysteine	3,851 ± 697	4,197 ± 892
Trolox	2,460 ± 809	5,741 ± 3,396
Resveratrol	2,683 ± 373	3,941 ± 852
MitoQ	7,447 ± 197	54,090 ± 3,876
Bezafibrate	1,233 ± 585	1,485 ± 340

ACAD9 deficient fibroblasts were exposed to 1,000 µM N-acetylcysteine, 1,000 µM Trolox (hydrosoluble analogue of vitamin E), 5 µM resveratrol, 200 nM mitoQ or 600 µM bezafibrate during 24 h before superoxide production measurement.

Supplementary Table S3. Mutations and clinical signs of ND6, NDUFV1 and ACAD9 deficient patients.

Patients	Defective protein	Genotype	Clinical signs
ND6	ND6	Heteroplasmic mutation on mtDNA at position 14459	Early onset developmental delay, seizures, lactic acidosis
NDUFV1	NDUFV1	c.611A>G (p.Y204C) c.616T>G (p.C206G)	Ataxia, bilateral ptosis, and ophthalmoplegia, lactic acidosis
ACAD9	ACAD9	c.1553G>A (p.R518H) c.1553G>A (p.R518H)	Severe cardio-myopathy, death at 4 months of life
